# Integrating Engineering, Manufacturing, and Regulatory Considerations in the Development of Novel Antivenoms

**DOI:** 10.3390/toxins10080309

**Published:** 2018-07-31

**Authors:** Andreas Hougaard Laustsen, Netty Dorrestijn

**Affiliations:** 1 Department of Biotechnology and Biomedicine, Technical University of Denmark, DK-2800 Kongens Lyngby, Denmark; 2 Utrecht Center for Affordable Biotherapeutics, Department of Pharmaceutical Sciences, Utrecht University, 3584 CG Utrecht, The Netherlands; j.dorrestijn@uu.nl

**Keywords:** snakebite envenoming, antivenom, monoclonal antibodies, antivenom manufacture, antivenom development, next-generation antivenom, antivenom regulation

## Abstract

Snakebite envenoming is a neglected tropical disease that requires immediate attention. Conventional plasma-derived snakebite antivenoms have existed for more than 120 years and have been instrumental in saving thousands of lives. However, both a need and an opportunity exist for harnessing biotechnology and modern drug development approaches to develop novel snakebite antivenoms with better efficacy, safety, and affordability. For this to be realized, though, development approaches, clinical testing, and manufacturing must be feasible for any novel treatment modality to be brought to the clinic. Here, we present engineering, manufacturing, and regulatory considerations that need to be taken into account for any development process for a novel antivenom product, with a particular emphasis on novel antivenoms based on mixtures of monoclonal antibodies. We highlight key drug development challenges that must be addressed, and we attempt to outline some of the important shifts that may have to occur in the ways snakebite antivenoms are designed and evaluated.

## 1. Introduction

Each year, snakebite envenoming exacts a death toll of more than 100,000 victims and maims more than 400,000 others [1]. In 2017, the World Health Organization (WHO) included snakebite envenoming on its list of neglected tropical diseases (NTDs) [2] and initiated the process of developing a global strategy for its prevention and treatment. Although an urgent necessity exists to increase the availability of existing antivenoms, to create more awareness of snakebite envenoming, and to train local healthcare personnel in treating snakebite in the affected regions [3], there is also a pressing need for innovation in the antivenom field. Since Césaire Auguste Phisalix, Gabriel Bertrand, and Albert Calmette simultaneously described the use of heterologous antivenom serotherapy in 1894 [4], limited developments have been introduced to the field of snakebite envenoming therapy, where animal-plasma-derived immunoglobulins (or fragments thereof) remain the mainstay of treatment [5]. To introduce novel therapeutic solutions in this field, the affordability of the final antivenom product is key [6]. Antivenom researchers should thus implement careful considerations on how to reduce both development and manufacturing costs. Cost reduction can be achieved through minimization of development risks, which in turn will improve the translation of novel experimental antivenoms into approved products that enter the clinic [7].

When the lab work for the scientist often ends, namely with publication of a lead identification for a certain target, the critical development process starts. The costs associated with this process dwarf any cost incurred in preclinical research and development, and a failure in clinical trials will have financially detrimental effects on an antivenom development program. It is thus essential to evaluate new scientific advances in the light of the risks associated with safety, regulatory approval, late stage development, and manufacture. Additionally, it is worth thoughtful evaluation regarding how compatible a novel antivenom product is with existing manufacturing and distribution infrastructure, as these aspects will have large implications on adaptability.

**Figure 1:**
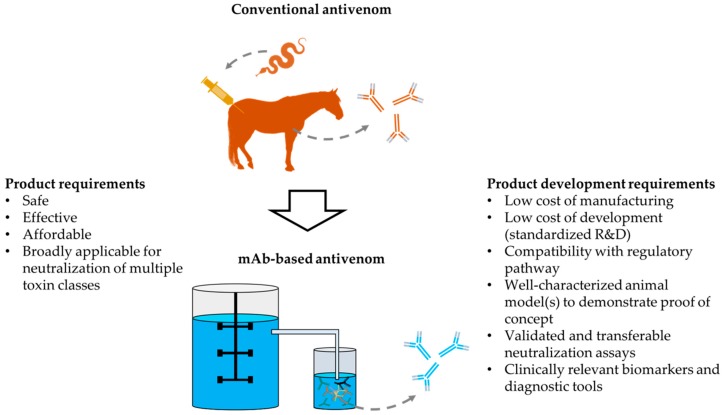
Schematic overview of the specific product requirements for novel antivenoms and the requirements that determine their ability to be developed.

Here, we wish to highlight important market, regulatory, development, and manufacturing aspects that are often neglected in the academic literature. In addition to safety and efficacy, we thus wish to call for integrating engineering, manufacturing, and regulatory considerations in the evaluation of novel antivenoms and the research programs necessary for these products (Figure 1).

## 2. Considerations for Novel Antivenoms and their Markets

The production costs for (equine) blood-derived immunoglobulins is increasing in general, and treatment or prophylaxis with blood-derived Igs in low and middle-income countries (LMICs) is under pressure [8]. This is not only the case for therapies against snakebite envenoming, but also for rabies, tetanus, and diphtheria prophylaxis. Therefore, in 2017, the WHO convened a meeting to discuss ways to explore how to replace of blood-derived immunoglobulins (Igs) for NTDs by monoclonal antibodies (mAbs) [9]. The specific advantages of mAbs over polyclonal Igs include standardized manufacturing processes, a globally large production capacity, and the possibility of producing these antibodies through cell cultivation according to good manufacturing practices (GMP). These attributes may improve scalability and decrease costs, thus improving access to and supply of safe alternatives to polyclonal Igs [9,10,11]. In animal models, numerous specific mAbs have already demonstrated their efficacy against snake venom toxins and even whole venom [12,13,14,15,16,17,18], which demonstrates that the discovery and development of mAb-based therapeutics against snakebite envenoming is indeed possible (a comprehensive overview of examples can be found in the reviews [5,19,20,21]).

A major part of the cost for therapeutic mAbs lies in the development phase. This phase also implicates the greatest risk and uncertainty, which is important to control to keep cost at a minimum [7]. Shared product development in public–private partnerships (PPPs) offers an alternative approach to conventional drug discovery by bringing together public partners (research programs in academia, governmental organizations such as the WHO, not-for-profit private sector partners, and philanthropists) with the for-profit private sector, such as pharmaceutical or biotech companies. In these constellations, cost is shared among the partners, which reduces the development costs for the new drug products, allowing for lower pricing for the benefit of the end payer (typically a public partner). Such constellations have previously been successful in bringing malaria vaccines to the market [22].

The global antivenom market, including antivenoms against scorpions and spiders, was in 2016 valued at USD 1.17 billion and is expected to grow to just under USD 1.58 billion by 2022 [23]. The snakebite antivenom market is segmented by geographical areas and the snake species present in these areas. The Australian and North American market represent the lion’s share of the global antivenom market due to the establishment of poison control centers and healthcare facilities in these regions, in addition to the willingness of payers to reimburse antivenom costs in the healthcare sector. However, the major snakebite burden exists in the Middle East, Africa, Asia, Oceania, and Latin America, where snakebite envenoming is a disease related to poverty [24,25,26,27]. This complicates the forecasting of formal market sizes for snakebite antivenom for LMICs due to reimbursement uncertainties. Nevertheless, a clear need for antivenom exists in these regions, where between 1.8 and 2.7 million people each year are afflicted with snakebite envenoming [1].

Plasma-derived antivenoms are manufactured to be specific against certain snake species and thus have specific regional markets based on the geographical occurrence of the snake species, whose venoms can be neutralized by given antivenoms (potentially including other snake species for which the antivenom is paraspecific). The fundamental reason for this is that antivenoms comprise antibodies that are raised in direct response to the venoms included in the immunization mixture employed in their manufacture. This has the implication that venom compositions have a determining role for the immunological response raised in the production animal, and therefore affect the final antibody composition of the antivenom [28]. This situation is different for future novel antivenoms based on specifically selected mAbs. Here, antibodies are discovered and carefully selected against specific isolated toxins using biotechnological methods [29] and guided by omics technologies [21] (particularly toxicovenomics [30,31,32]), which may possibly also offer the possibility of developing antibodies that have broad specificity against entire toxin subfamilies [28]. This may allow for novel rational design strategies that provide the possibility of developing mAb-based antivenoms with the ability to neutralize venoms from a broader range of species across a larger geographic area, as the antibody compositions of such antivenoms can be tailored to specifications. Therefore, the market sizes for mAb-based antivenoms cannot be estimated in exactly the same way as plasma-derived antivenoms, but instead require a new view. In this regard, it is likely that the market sizes for mAb-based antivenoms may supersede those of plasma-derived antivenoms due to the possibility of expanded species coverage.

## 3. Development and Manufacturing Aspects

For any molecule to be considered relevant as a therapeutic, it must be manufacturable at large scale and at a cost that is within the limits of what the end payer is willing and able to cover. For many diseases that affect high-income countries, the requirement for low-cost manufacturing may not be as critical as for diseases that mainly affect impoverished nations. Unfortunately for snakebite envenoming, most victims live in poor, rural areas of the tropics, and snakebite has been shown in many places to be directly correlated with poverty and lack of proper access to healthcare [24,25,26]. This puts a strong demand on the cost-effectiveness for new snakebite envenoming therapies [33] and may indeed prevent non-standard therapeutic molecules requiring specialized and intricate manufacturing processes from ever being able to enter the market. This implies that “stand-alone leads” that only target one or few toxins within the same toxin family, and which cannot easily be modified to target other toxin families may not deserve further investigation than that which pure academic curiosity allows for, as it is simply not economically feasible to develop a specialized manufacturing process for “stand-alone leads”. As an example of a cost-competitive manufacturing approach that could be useful in the field of recombinant antivenoms, the Sympress technology [34] has successfully been employed to express oligoclonal mixtures of human IgGs that have entered clinical trials for indications within oncology and autoimmunity [35,36,37].

To evaluate whether or not a given type of molecule could indeed be cost-effective and cost-competitive with existing antivenoms, it is important to fully understand the cost of goods sold (COGS) of the molecule. In much academic literature in the field of next-generation antivenom research, claims of the low cost of a potential pharmaceutical candidate are solely based on the (presumed) cost of upstream processes (synthesis and/or fermentation). Downstream processes, ‘fill and finish’, and quality assurance (QA) are often not accounted for, despite that these parts of the manufacturing process are the most expensive for many pharmaceutical products [10,38,39,40]. Other claims that find their way into the academic literature include that protein expression by microbial/prokaryotic fermentation processes is much less expensive than mammalian cell cultivation. Although this may indeed be true for certain processes at large scale, such claims lack nuance and seem to neglect that microbial/prokaryotic fermentation processes often require extensive chemical engineering efforts and a more sophisticated manufacturing setup (including steel tanks fitted with cooling and expensive ‘cleaning in place (CIP)’ procedures [39]), which in turn requires significant upfront capital investment [41]. In contrast, mammalian cell cultivation is nowadays routinely performed using simple, single-use disposable equipment that does not require an advanced chemical engineering setup, and which minimizes the downtime between batches [42,43]. Additionally, as less chemical engineering and development is needed, the time-to-market may also be shorter for therapeutics manufactured by routine mammalian cell cultivation, which may also have an impact on cost due to the lowering of indirect costs [41,44]. When evaluating the cost of manufacturing of a pharmaceutical candidate, these aspects combined with the volume of production may have a large impact [10,39], and proper cost simulations are necessary to select the most appropriate manufacturing strategy. As a rule of thumb, for smaller production volumes, microbial/prokaryotic fermentation processes may have difficulty in competing on cost with mammalian cell cultivation due to increased costs for process development and higher indirect costs of production resulting from a more advanced manufacturing setup.

As a final word on the development and manufacturing processes for novel antivenoms, it is relevant to consider molecules for which quality control (QC) is easy to perform and validate, and where manufacturing processes are easy to transfer to low-cost manufacturing sites. Again, this may drastically lower the feasibility of investigating the use of non-standard therapeutic molecules for antivenom purposes.

## 4. Clinical and Regulatory Aspects

To support governments and healthcare systems in tackling the challenge of snakebite envenoming, the WHO has developed guidelines for the production, control, and regulation of antivenom immunoglobulins [45]. However, when it comes to developing fundamentally novel antivenoms not derived from animal plasma, a number of existing topics in these guidelines become redundant and new topics need to be developed for products with a fundamentally different nature, such as antivenoms based on mAbs. As a comparable example for respiratory syncytial virus (RSV), the WHO’s Department of Immunization, Vaccines and Biologicals has developed both a research and development technology roadmap describing the priority activities for the development, testing, and use of products for the treatment of RSV for LMICs [46], as well as having defined the preferred product characteristics for RSV vaccines [47].

To facilitate the introduction of novel antivenom products, a need also exists for developing and incorporating robust diagnostic methodologies that allow for correct identification of the snake species responsible for a given bite. Such methodologies could either be based on novel diagnostic tests (venom/toxin detection kits) or scoring systems and algorithms that allow physicians to differentiate between e.g., cytoxic/hemotoxic viper envenomings and elapid neurotoxic envenomings based on clinical manifestations [48]. Having reliable diagnostic methodologies is an absolute prerequisite for the planning of robust clinical trials and patient stratification. On the other hand, the need for pharmacokinetic studies may be reduced if novel antivenom products are to be based on mAbs. After 25 years of clinical experience with therapeutic mAbs, the pharmacokinetic behavior in both adults and children is often predictable and well-known [49], but must still be evaluated for new mAb-based therapeutics.

Demonstrating efficacy and safety in clinical trials is difficult, expensive, and time-consuming. Therefore, the need for venom/toxin-specific well-characterized in vitro assays and animal models to demonstrate proof of concept and even predict efficacy before entering into clinical trials is crucial [50]. To keep the development phase affordable for novel non-plasma-derived antivenoms, a shift towards more extensive early research and a reduction of late (clinical) stage activities should be implemented. Developing a completely novel antivenom product will also create an opportunity for researchers to develop and validate new in vitro assays that might replace or reduce animal studies [51].

An accelerated regulatory approval process for a mAb-based snakebite antivenom will be challenging. National regulatory agencies (NRAs) in the LMICs should ensure that available products, whether imported or locally manufactured, are of high quality, safe, and efficacious, and should thus ensure that manufacturers adhere to approved standards regarding quality assurance and GMP. To expedite regulatory processes, facilitated regulatory pathways for new medicines in emerging economies have been implemented [52]. The European Medicines Agency (EMA) and the WHO aim to facilitate the review process by making recommendations for approval. The EMA facilitates registration for products that will be used outside the EU through the EMA Article 58 procedure, and the WHO has a Prequalification Program for LMICs, enabling registration as well as procurement [53]. Taken together, for a novel treatment modality to be used mainly in LMICs, it is crucial to start discussions with all parties involved in the regulatory process as early as possible. Special requirements to enable the clinical and regulatory process, such as specific assays or biomarkers for preclinical development and diagnostic tools to stratify snakebite victims (e.g., by identifying the perpetrating snake species responsible for a given envenoming case) to optimize early medical treatment, need to be considered as action points in the roadmap for technical development. Furthermore, depending on the specific action of a novel antivenom product, such assays and biomarkers should be implemented in early research and development. Following this approach, the traditional and expensive linear development process (phases I to IV) may even be replaced with a question-driven approach to develop an innovative and affordable therapy against snakebite envenoming [54].

Finally, it may be highly relevant to interact both with the healthcare systems in countries with weak economies and not-for-profit organizations to secure political backing and economic support to subsidize snakebite antivenoms for them to be affordable for impoverished victims [3,25].

## 5. Conclusions and Recommendations

An urgent need exists for the provision of safe, affordable, and effective snakebite antivenoms to victims worldwide. Part of a long-term sustainable solution may be to harness biotechnological approaches for the development of novel antivenom products. These may possibly be based on mixtures of human monoclonal antibodies that could directly replace the equine polyclonal antibodies currently used in antivenoms. For a transition from conventional plasma-derived antivenoms to novel mAb-based antivenoms to happen, a need presents itself not only to evaluate the efficacy and safety of new products, but also to evaluate the possibility for such new products to be developed. To facilitate this, we recommend that engineering, manufacturing, and regulatory aspects are taken into consideration in the research and development of novel snakebite antivenoms, as these aspects may be either a key to success or detrimental to next-generation antivenom development plans.
